# Minimally invasive needle tenotomy vs. platelet rich plasma injection in the treatment of chronic elbow epicondylitis

**DOI:** 10.1016/j.jseint.2024.08.183

**Published:** 2024-08-28

**Authors:** Chantal T. Nguyen, Michelle H. Lee, Matthew W. Kaufman, Yue Meng, Jyotsna A. Koduri, Geoffrey Abrams, Emilie V. Cheung, Michael T. Freehill, Eugene Y. Roh

**Affiliations:** Division of Physical Medicine & Rehabilitation, Department of Orthopedic Surgery, Stanford University, Stanford, CA, USA

**Keywords:** Elbow epicondylitis, Platelet-rich plasma injection, Tenotomy, Pain, Function, Imaging

## Abstract

**Background:**

Medial and lateral epicondylitis, characterized by repetitive microtraumas to common flexor and extensor tendons, respectively, are common causes of elbow pain in adults. Though symptoms are generally self-limiting, 10% of cases are refractory to conservative management, persisting for greater than 18 months, and leading to surgery, which can have increased risk of complications. There is minimal data on sustained pain relief and functional benefit for newer nonsurgical management options, such as minimally invasive needle tenotomy (MINT), and platelet-rich plasma (PRP) for chronic elbow epicondylitis. Additionally, no previously established correlation exists between magnetic resonance imaging (MRI) severity of chronic epicondylitis with pain and functional improvement in MINT- and PRP-treated patients.

**Methods:**

A retrospective review of 51 adults (n = 23 for MINT; n = 28 for PRP) was conducted to investigate long-term outcomes in pain relief (via visual analog scale or VAS) and improvements in upper extremity function (via quick disability of the arm, shoulder, and hand questionnaire or qDASH). These outcomes were correlated with radiographic evidence of epicondylitis severity, assessed by the grade of epicondylitis and percentage thickness of tendon tears.

**Results:**

There were significant improvements in pain (VAS), but no significant differences in function (qDASH) following MINT and PRP. On average, VAS score improved by 2.6 (*P* < .001) post-MINT and by 3.8 (*P* < .001) post-PRP combined for all follow-up time points. No adverse events were reported over the entire study. A significantly higher percentage of patient-reported pain relief was noted post-MINT at all follow-up time points. VAS and qDASH outcomes post-MINT and post-PRP were not correlated with the initial MRI severity of epicondylitis.

**Conclusions:**

There are multifactorial benefits of both MINT and PRP as safe, nonopen surgical modalities that can be used, despite MRI severity, to provide sustained pain relief for patients with refractory elbow epicondylitis.

Elbow pain is a common upper extremity complaint seen in both primary care and sports medicine clinics, but the specific drivers of pain can be multifactorial.^7^ Though pathology can affect the elbow joint, bursae, nerves, or tendons, the most common etiology of elbow pain involves tendinopathies, namely lateral epicondylitis (LE) and medial epicondylitis (ME). Both conditions are hypothesized to be from excessive microtraumas due to repetitive wrist or forearm movement, leading to degenerative tendinosis, increased molecular inflammation, and increased focal pain typically at the lateral epicondyle, where the extensor tendons attach or at the medial epicondyle, where the flexor tendons attach.^3^^,^^15^ LE and ME primarily affect adults between 35 and 55 years old, with a prevalence affecting 1%-3% of the population annually.^3^^,^^20^

Though the majority of cases are self-limiting and resolve with conservative treatment, including nonsteroidal anti-inflammatory medications, bracing, and/or physical therapy, there is a 10% incidence of refractory cases lasting for greater than 18 months and causing significant functional limitations, including lost time from work and inability to engage in routine daily or recreational activities.^3^^,^^15^^,^^23^ One in ten patients who fail conservative management ends up pursuing surgical intervention.^19^ Thus, a variety of methods have been evaluated for the treatment of refractory LE or ME. There has been insufficient evidence to support or refute the efficacy of surgical management for refractory LE or ME,^4^ nor to support a specific, preferred nonsurgical treatment method, such as steroid injections, iontophoresis, prolotherapy, or bracing.^3^^,^^23^ However, surgical management, which aims to release the common flexor or extensor tendons at the elbow, can lead to complications, such as ligamentous instability, septic arthritis, neuroma formation, and delays in return to work or activity.^1^ Pursuing nonsurgical options may be advantageous for decreasing symptom burden, minimizing risk, and expediting return to work.

Two such nonsurgical options are platelet-rich plasma (PRP) injections and percutaneous tenotomy with needle fenestration. PRP injections involve collecting whole blood from patients, centrifuging the components to isolate the platelet-rich portion, and injecting it into diseased tendon to promote healing via release of growth factors.^2^^,^^17^ Percutaneous needle tenotomy involves creating fenestrations in pathologic tendon to break up scar tissue and promote tissue healing. One approach of percutaneous tenotomy uses ultrasonic energy with a Tenex device (Tenex Health Inc, Lake Forest, CA, USA).^2^^,^^8^ Percutaneous needle tenotomy has demonstrated safety and effectiveness in pain relief for refractory LE and ME, with the added benefit of shorter recovery times, fewer complications, and lower cost when compared to surgical management.^12^^,^^15^^,^^16^^,^^26^^,^^27^ In addition, another type of tenotomy called Tenjet (HydroCision Inc., North Billerica, MA, USA), which involves the use of high-velocity saline, with a suction at the needle tip under ultrasound guidance to remove pathologic tendon, has been investigated in as a possible treatment option for chronic elbow epicondylitis. One recent study reports benefit in physical function and pain relief following percutaneous tenotomy for LE refractory to conservative management,^7^ but no comparison of MINT to PRP has been performed. It is also unclear if the severity of ME or LE, as determined by radiographic MRI evaluation and degree of pain, impacted improvement in tenotomy outcomes,^5^ or if patients had long-term symptom resolution.

This study investigates the pain relief and safety outcomes between minimally invasive needle tenotomy (MINT) and PRP injections in the management of refractory elbow epicondylitis as well as the correlation between elbow tendon findings on MRI and nonsurgical treatment outcomes for elbow epicondylitis. It is hypothesized that both MINT and PRP can safely lead to long-term pain relief and improvements in function in chronic elbow epicondylitis, especially in more radiographically severe cases.

## Methods

A retrospective cohort chart review of 200 patient records was conducted at a single, large academic orthopedic sports and upper extremity clinic. The inclusion criteria for study participants included patients who were above 18 years of age with clinical presentation and MRI findings consistent with lateral or medial elbow epicondylitis who had refractory symptoms for at least three months despite at least one conservative treatment option. Each of the patients was offered either PRP injection or MINT, performed by a single triple-boarded physician certified in Physical Medicine and Rehabilitation, Sports Medicine, and Internal Medicine specializing in orthobiologics. Ultimately, it was a patient’s decision to undergo PRP or MINT though insurance considerations could play a role in which option was selected, as MINT has the possibility of being covered by insurance, whereas PRP is typically an out-of-pocket expense.

PRP injection was prepared by drawing approximately 30cc of whole blood from the antecubital fossa of the arm. The blood was spun in a centrifuge Under ultrasound guidance, a 25G 1 1/2 inch needle was used to deliver approximately 3cc of PRP at the injured lesion of the common extensor or flexor tendon.

The MINT procedure was performed in the outpatient surgery center with the patient in the supine position. Minimal sedation was used as needed. A #11 blade was used to make a 2mm incision. The MINT débridement instrument was inserted into this incision site and visualized with ultrasound. The tip of the instrument was directed to the pathologic tendon area, and then the tip was activated. SteriStrips (3M Healthcare, St. Paul, MD, USA) or Dermabond (Johnson & Johnson, New Brunswick, NJ, USA) were used to close the incision site. Tegaderm (3M Healthcare, St. Paul, MD, USA) was then used to seal the dressing.

The exclusion criteria included recipients of reimbursement (either disability or worker’s compensation) and recipients with active litigation relevant to their elbow condition. Fifty-one patients were identified and included in the study. Clinical data including total symptom duration, follow-up frequency, prior treatments, prior medications, and dominant hand involvement for each patient were collected from the electronic medical record. Postprocedure outcome surveys were collected by treating physicians and clinic staff during each patient’s clinic visit, remote telehealth visit, or via telephone encounter. Outcome surveys for subjective assessment of pain via the visual analog scale (VAS) and function via the quick disability of the arm, shoulder, and hand (qDASH) questionnaire were collected at one, three, and six months. For the subgroup analysis, elbow MRI imaging was reviewed and classified by severity as determined by the reading radiologist, with classification system, including mild, moderate, or severe epicondylitis. An additional MRI classification tool involved assessing the severity of tear, graded by no tear, <50% partial thickness tear, >50% partial thickness tear, or full-thickness tear was also used.

The demographic and clinical differences between PRP and MINT groups were analyzed using two-sample t-tests, Mann–Whitney tests, Chi-square tests, and Fisher’s exact tests, depending on assumption satisfaction. Multivariable mixed-effects linear and logistic regression models were used to analyze post-procedural outcomes, including changes in pain levels, pain relief as a percent, odds of significant or complete improvement, odds of adverse events, and odds of additional procedures. These models were subsequently repeated, adding MRI severity classifications (mild, moderate, and severe) to evaluate its effect on outcomes. Patient identification numbers were included as a random variable to account for multiple years of follow-up. Pairwise comparisons report false discovery rates-adjusted *P* values and use a two-sided level of significance of 0.05. All analyses were completed using RStudio version 1.3.1093-1 (Boston, MA, USA).

## Results

Of the 51 patients who met the inclusion criteria for the study, 23 of these patients underwent MINT and 28 underwent a PRP injection. Fifty-one out of 51 patients completed the postprocedure outcome surveys (100% follow-up rate). The mean age of the MINT group was 44.3 years, which was significantly lower than the mean age of the PRP group at 52.3 years (*P* = .006). Otherwise, demographic analysis revealed no significant differences in body mass index, gender, race, or ethnicity (Table I).

**Table I:** Demographic evaluation of participants undergoing MINT vs. PRP for elbow epicondylitis.

	MINT (n = 23)		PRP (n = 28)		*P*-value
Age (mean, SD)	44.3	7.1	52.3	12.4	.006∗
Body mass index(median, IQR)	25.3	23.2-32.0	24.8	22.2-26.8	.159
Gender (n, %)					
Female	6	26%	8	29%	>.999
Male	17	74%	20	71%	
Race (n, %)					
American Indian	1	5%	0	0%	.200
Asian	2	11%	7	28%	
White	16	84%	18	72%	
Ethnicity (n, %)					
Hispanic/Latino	5	22%	5	18%	.739
Not Hispanic/Latino	18	78%	23	82%	

*MINT*, minimally invasive needle tenotomy; *PRP*, platelet-rich plasma; *IQR*, interquartile range; *SD*, standard deviation.

*P*-value of <.05 demonstrates statistically significant difference between the MINT group and PRP group.

∗ Demonstrates statistically significant differences.

Baseline clinical data including symptom duration (in months), follow-up frequency, prior conservative management options, prior medications, and dominant hand involvement demonstrated no statistical difference in any outcome between the two treatment groups (Table II). MRI classification of elbow epicondylitis revealed no significant differences in injury severity between the MINT and PRP groups, using both radiologist description and MRI classification of tear size (Table III).

**Table II:** Baseline clinical data including symptom duration, follow-up frequency, prior treatments, prior medications, and dominant hand involvement for MINT vs. PRP groups.

	MINT (n = 23)		PRP (n = 28)		*P*-value
Symptom duration (median # of mo, IQR)	8	4-12	11.5	5.3-24	.178
# of follow-up (Mean, SD)	2.3	1.3	2.14	0.77	.610
Total follow-up duration (mean # of d, SD)	297.30	396.99	229.55	289.17	.528
Prior treatments (n, %)					
Physical therapy	17	74%	18	64%	.759
Bracing	18	78%	17	61%	.632
Medication	19	83%	16	57%	.400
Steroid injection	6	26%	11	39%	.648
Shock wave	1	4%	0	0%	.648
Acupuncture	3	13%	1	4%	.632
Massage	2	9%	3	11%	>.999
HEP	8	35%	2	7%	.240
Prior medications (n, %)					
NSAIDs	17	74%	19	68%	>.999
Topical Nitrate	1	4%	1	4%	>.999
Opioids	1	4%	0	0%	>.999
Affects dominant hand (n, %)					
No	2	10%	3	14%	>.999
Yes	19	90%	18	86%	

*NSAIDs*, nonsteroidal anti-inflammatory medications; *MINT*, minimally invasive needle tenotomy; *PRP*, platelet-rich plasma; *HEP*, home exercise program; *IQR*, interquartile range; *SD*, standard deviation.

*P*-value of <.05 demonstrates statistically significant difference between the MINT group and PRP group.

**Table III:** MRI classification of severity of elbow epicondylitis had no statistically significant differences in injury severity between the MINT and PRP groups.

	MINT		PRP		*P*-value
MRI classification (n, %)					
Mild	3	16%	8	50%	.065
Moderate	15	79%	8	50%	
Severe	1	5%	0	0%	
MRI finding (n, %)					
No tear	1	5%	5	19%	.288
Partial thickness tear (<50%)	3	14%	7	27%	
Partial thickness tear (>50%)	6	29%	5	19%	
Partial thickness (no grading)	11	52%	9	19%	

*MRI*, magnetic resonance imaging; *MINT*, minimally invasive needle tenotomy; *PRP*, platelet-rich plasma.

**Figure 1:**
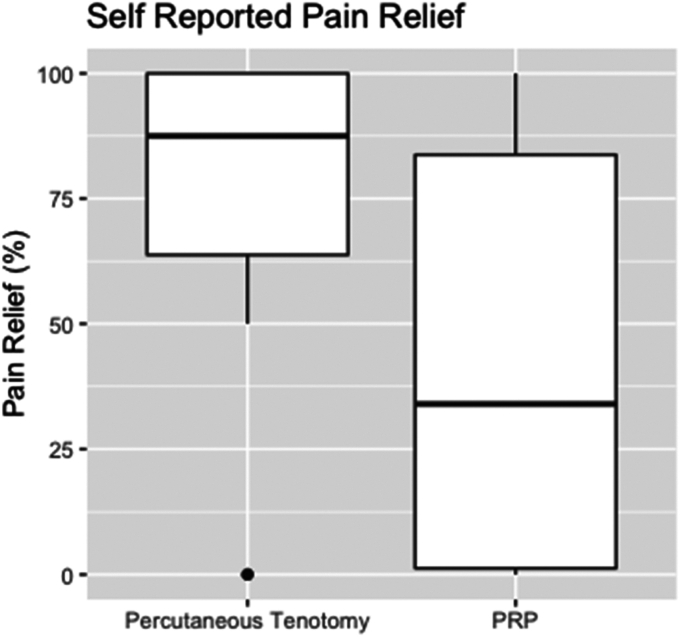
Comparison of self-reported pain relief between PRP and MINT groups for elbow epicondylitis. *MINT*, minimally invasive needle tenotomy; *PRP*, platelet-rich plasma.

There were, however, statistically significant decreases in overall elbow pain postprocedure, measured by VAS at one (improved by 3 points, *P* = .008 for PRP; improved by 2.56 points, *P* = .033 for MINT) and three months (improved by 2.95 points, *P* = .026 for PRP; improved by 6 points, *P* = .011 for MINT) (Table IV). Including all time points, VAS improved for both MINT (improved by 2.6 points, *P* < .001) and PRP groups (improved by 3.8, *P* < .001) (Table IV). When comparing self-reported pain relief by percentage, the MINT group demonstrated greater self-reported pain relief percentage than that of the PRP group (*P* = .032; Table V and Fig. 1); however, there was no statistical difference for changes in VAS score at long term (Table V). Of note, data at the six-month follow-up for many patients were incomplete, so improvements in VAS were not able to be determined accurately for that time point.

**Table IV:** Changes in pain (VAS) pre- and post-treatment with either MINT or PRP injection for elbow epicondylitis, divided by all time points, at 1-mo postprocedure, and at 3-mo post-procedure.

PRP	Change in pain VAS score				1 mo change				3 mo change			
	Estimate	Lower 95%	Upper 95%	*P*-value	Estimate	Lower 95%	Upper 95%	*P*-value	Estimate	Lower 95%	Upper 95%	*P*-value
Treatment (Post vs. Pre)	−2.60	−3.82	−1.45	<.001	−3.00	−5.02	−0.98	.008∗	−2.95	−5.48	−0.43	.026∗
Follow-up length (Mo)	−0.03	−0.09	0.04	.482								
Age (y)	0.03	−0.03	0.08	.38								
Symptom duration (mo)	0.02	0.03	0.07	.423								
Number of prior treatments	−0.03	−0.50	0.46	.928								

MINT	Change in pain VAS score				1 mo change				3 mo change			
	Estimate	Lower 95%	Upper 95%	*P*-value	Estimate	Lower 95%	Upper 95%	*P*-value	Estimate	Lower 95%	Upper 95%	*P*-value
Treatment (Post vs. Pre)	−3.80	−5.14	−2.52	<.001	−2.56	−4.88	−0.23	.033∗	−6.00	−9.93	−2.07	.011∗
Follow-up length (mo)	−0.003	−0.07	0.07	.94								
Age (y)	−0.004	−0.12	0.11	.955								
Symptom duration (mo)	−0.002	−0.11	0.10	.969								
Number of prior treatments	1.11	0.28	1.94	.029								

*VAS*, visual analog scale; *MINT*, minimally invasive needle tenotomy; *PRP*, platelet-rich plasma.

*P*-values of <.001 demonstrate statistically significant decreases in overall elbow pain postprocedure for both MINT and PRP groups.

∗ Demonstrates statistically significant differences.

**Table V:** Percentage of pain relief following MINT vs. PRP for elbow epicondylitis demonstrated statistically significant self-reported pain relief in the MINT group but not the PRP group.

	Pain relief as a percent			
	Estimate	Lower 95%	Upper 95%	*P*-value
Treatment (PRP vs. MINT)	−39.00	−72.99	−5.01	.032
Follow-up length (mo)	0.99	−0.29	2.27	.140
Age (y)	−0.90	−2.30	0.50	.216
Symptom duration (mo)	0.84	−0.12	1.80	.096
Number of prior treatments	−4.15	−15.71	7.40	.487

	Change in pain VAS score			
	Estimate	Lower 95%	Upper 95%	*P*-value
Treatment (PRP vs. MINT)	1.93	−0.28	4.13	.097
Follow-up length (mo)	−0.05	−0.12	0.01	.128
Age (y)	−0.01	−0.11	0.09	.815
Symptom duration (mo)	−0.06	−0.15	0.03	.208
Number of prior treatments	0.32	−0.59	1.22	.496

*VAS*, visual analog scale; *MINT*, minimally invasive needle tenotomy; *PRP*, platelet-rich plasma.

However, there was no statistical difference for changes in VAS score at follow-up in either group.

Additional comparison analyses, including assessment of complete symptom resolution, adverse events, and additional procedures, were performed; there was no statistical difference between PRP and MINT for any of these outcomes (odds ratio 0.32; *P* = .099) (Table VI). However, adjusting for treatment groups, the odds of significant or complete improvement were 1.51 times higher with each additional month of follow-up (*P* = .008; Table VI). There was additionally no difference in outcome (change in VAS score, pain relief as a percent, complete or significant symptom resolution, or adverse events) when comparison was made between various severity subgroups based on MRI findings (Table VII).

**Table VI:** Analysis of complete symptomatic improvement, adverse events, and requirement for additional procedures showed no statistically significant difference between MINT and PRP groups.

	Significant or complete improvement		Adverse events		Additional procedures	
	Odds ratio	*P*-value	Odds ratio	*P*-value	Odds ratio	*P*-value
Treatment (PRP vs. MINT)	0.32	.099	0.93	.979	1.63	.76
Follow-up Length (mo)	1.51	.008	1.01	.985	2.05	.126
Age (y)	0.99	.737	0.96	.881	0.99	.916
Symptom duration (mo)	1.03	.215	0.75	.550	0.96	.478
Number of prior treatments	0.77	.278	0.76	.844	1.38	.595

*MINT*, minimally invasive needle tenotomy; *PRP*, platelet-rich plasma.

**Table VII:** Assessment of change in VAS score, pain relief as a percent, complete or significant symptom resolution, or adverse events based on MRI severity.

Outcome	Comparison	Estimate (mean difference)	Lower 95%	Upper 95%	*P*-value
Change in pain	Mild vs. Severe	0.4	−2.4	3.2	.778
	Moderate vs. Severe	0.2	−7.0	7.5	.950
Pain relief as a percent	Mild vs. Moderate	−9.2	−45.5	27.1	.634

Outcome	Comparison	odds ratio	Lower 95%	Upper 95%	*P*-value
Significant or complete improvement	Mild vs. Moderate	0.82	0.16	4.05	.804
Adverse events	Mild vs. Moderate	0.81	0.03	25.11	.906

*VAS*, visual analog scale; *MRI*, magnetic resonance imaging.

## Discussion

Our findings support follow-up data for pain relief post-MINT and PRP in chronic elbow epicondylitis, with greater symptomatic self-reported pain relief following MINT over time. Options for management of chronic lateral or ME include both surgical and nonsurgical modalities, but few studies have examined a head-to-head comparison of long-lasting improvements in pain between tenotomy and PRP injections for elbow epicondylitis. A recent retrospective study focused on ultrasonic tenotomy vs. PRP, in only chronic LE over twelve weeks, showed no difference in pain between the two modalities, but there are no studies comparing this to PRP in chronic ME as well.^18^ A comparative study from 2019 examined Tenex vs. PRP for refractory medial and LE and found significant improvements in pain (VAS) and function (quick disability of the arm, shoulder, and hand and EuroQOL-5D scores), but no difference in pain relief or function between MINT and PRP.^3^ The average follow-up times were about 10-month postprocedure for the MINT group and 17-month postprocedure for the PRP group.

Our findings expound upon several unique factors, as there is no current study examining outcomes correlating with MRI findings between MINT and PRP for chronic medial and lateral elbow epicondylitis. When adjusting for MINT and PRP groups, there were increased odds of significant or complete improvement that was significantly (1.51 times) higher for each additional month of follow-up. Reported significant pain relief that improves with each additional month of follow-up can be attributed to possible molecular restructuring of the underlying diseased tendon via locally increasing concentration of growth factors to an injured area, which occurs in both PRP and MINT, though PRP is thought to be more symptom-modifying than structure-modifying.^3^

However, in our study, MINT may have led to additionally improved patient-reported percentage of pain relief given that this modality physically removes diseased tendon. Diseased tendon is characterized by loss of extracellular matrix structure through increased production of proteoglycans and glycosaminoglycans, decreased tissue/collagen elasticity, increased edema, and increased cell apoptosis in surrounding tendon fibers.^24^ The percutaneous tenotomy system operates via a high-pressure saline or an ultrasonic system that can mechanically evacuate pathologic aspects of tendon that is the pain generator, thus minimizing the downstream proinflammatory, destructive release of vascular endothelial growth factor, and optimizing micronutrient delivery for healthy tendon to predominate.^9^^,^^13^^,^^25^ This has profound implications given the chronic symptom burden and opportunity cost of tendinopathies. Given that elbow epicondylitis typically occurs as a result of repetitive microtraumas, individuals who do manual labor often are at higher risk, and their ability to return to work in a timely manner is inversely related to long-term disability and possible economic instability.^14^ Return to work can be influenced by whether the patient’s dominant hand is affected. In our patient population, the majority of symptoms affected the dominant hand (90% for MINT, 87% for PRP), so noninvasive treatment has the dual benefit for pain relief and expediting return to work. In our study, the MINT treatment group was younger (average 44.3 years vs. 52.3 years in the PRP group), and age-related correlation with loss of tendon elasticity could also account for this subjective improvement in pain over the PRP group.^11^ It is also interesting to note that though the MINT group was younger, this group had higher severity of elbow epicondylitis as seen via MRI than that of the PRP group but still had similar improvements in pain relief.

Because MRIs can be ordered in refractory cases of elbow epicondylitis, this study examined the relationship between MRI severity and grading of tendon tears, which has not been explored in the literature to date. The majority of sampled patients had MRI-reported mild-to- moderate elbow epicondylitis with partial thickness tears, with only one case of severe epicondylitis in the MINT group. Despite some differences in MRI severity and grading of tendon tears,^5^ there was no significant correlation observed between the degree of tendon tearing on MRI with post-MINT or PRP outcomes; this possibly suggests radiographic findings as a less important factor in predicting improvements in pain and function in nonsurgical management of elbow epicondylitis.^10^^,^^21^

There were no adverse events in both the MINT and PRP groups, which support their overall safety profile. Despite the theoretical risk of tendon rupture with MINT, given its mechanism of action, our study supports previous literature stating no long-term complications posttenotomy.^6^ It is important to note that the physician performing these procedures is an orthobiologics expert, so results should be considered in light of this factor.

There were several limitations to the study, many of which are inherent to conducting a retrospective review. First, there were variable follow-up times due to the nature of data collection via chart review, and the treatment was not randomized. There was also lack of a consistent MRI grading system, especially with possibly subjective qualifications of mild vs. moderate or moderate vs. severe elbow epicondylitis. We attempted to mitigate the subjectivity behind MRI grading by adhering to terminology as dictated from the reading radiologist, but there is room for more accurate radiographic descriptions of tendon pathology. Future studies would be aimed toward a prospective analysis to further corroborate findings and assess for any correlation between MRI findings and other nonsurgical treatment outcomes. In addition, though the literature has shown improved outcomes in pain relief from the use of leukocyte-rich PRP, this study assessed outcomes including leukocyte-poor PRP.^22^ In addition, given the evolving nature of MINT for use in the treatment of other types of tendinopathies, including the gluteus medius tendon and hamstring tendons, its widespread efficacy should be further explored.

## Conclusion

For refractory lateral and ME, irrespective of the initial radiographic severity on MRI, two safe, effective, and nonsurgical management options are MINT and PRP injections. Though reported pain relief as a percent is higher in the MINT group, both procedures can induce long-lasting pain relief and lead to more complete pain relief with each additional month of follow-up in refractory elbow epicondylitis.

## Acknowledgments

The authors would like to acknowledge their biostatistician, Nicole S. Pham, MPH.

## Disclaimers:

Funding: There were no outside sources of funding or grants for this study.

Conflicts of interest: The authors, their immediate families, and any research foundation with which they are affiliated have not received any financial payments or other benefits from any commercial entity related to the subject of this article.

## References

[1] Amroodi M.N., Mahmuudi A., Salariyeh M., Amiri A. (2016). Surgical treatment of tennis elbow; minimal incision technique. Arch Bone Jt Surg.

[2] Barnes D.E., Beckley J.M., Smith J. (2015). Percutaneous ultrasonic tenotomy for chronic elbow tendinosis: a prospective study. J Shoulder Elbow Surg.

[3] Boden A.L., Scott M.T., Dalwadi P.P., Mautner K., Mason R.A., Gottschalk M.B. (2019). Platelet-rich plasma versus Tenex in the treatment of medial and lateral epicondylitis. J Shoulder Elbow Surg.

[4] Buchbinder R., Johnston R.V., Barnsley L., Assendelft W.J., Bell S.N., Smidt N. (2011). Surgery for lateral elbow pain. Cochrane Database Syst Rev.

[5] Cha Y.K., Kim S.J., Park N.H., Kim J.Y., Kim J.H., Park J.Y. (2019). Magnetic resonance imaging of patients with lateral epicondylitis: relationship between pain and severity of imaging features in elbow joints. Acta Orthop Traumatol Turc.

[6] Chalian M., Nacey N.C., Rawat U., Knight J., Lancaster T., Deal D.N. (2021). Ultrasound-guided percutaneous needle tenotomy using Tenex system for refractory lateral epicondylitis; short and long-term effectiveness and contributing factors. Skeletal Radiol.

[7] Dakkak M., Patel V., King D., Genin J. (2023). Ultrasound-guided tenotomy for lateral epicondylitis with improves physical functional and decreased pain outcomes at 1 year: a case series review. JSES Int.

[8] Jacobson J.A., Kim S.M., Brigido M.K. (2016). Ultrasound-guided percutaneous tenotomy. Semin Musculoskelet Radiol.

[9] Kamineni S., Butterfield T., Sinai A. (2015). Percutaneous ultrasonic debridement of tendinopathy-a pilot Achilles rabbit model. J Orthop Surg Res.

[10] Kessler R.E., Day M.S., Tyler T.F., McHugh M.P., Bedford B.B., Lee S.J. (2022). Predictive value of magnetic resonance imaging in outcomes of nonsurgical treatment of lateral epicondylitis. JSES Int.

[11] Korcari A., Przybelski S.J., Gingery A., Loiselle A.E. (2023). Impact of aging on tendon homeostasis, tendinopathy development, and impaired healing. Connect Tissue Res.

[12] Lakhey S., Mansfield M., Pradhan R.L., Rijal K.P., Paney B.P., Manandhar R.R. (2007). Percutaneous extensor tenotomy for chronic tennis elbow using an 18G needle. Kathmandu Univ Med J.

[13] Liu X., Zhu B., Li Y., Liu X., Guo S., Wang C. (2021). The role of vascular endothelial growth factor in tendon healing. Front Physiol.

[14] Marom B.S., Ratzon N.Z., Carel R.S., Sharabi M. (2019). Return-to-Work barriers among manual workers after hand injuries: 1-year follow-up cohort study. Arch Phys Med Rehabil.

[15] Mattie R., Wong J., McCormick Z., Yu S., Saltychev M., Laimi K. (2017). Percutaneous needle tenotomy for the treatment of lateral epicondylitis: a systematic review of the literature. PM R.

[16] McShane J.M., Nazarian L.N., Harwood M.I. (2006). Sonographically guided percutaneous needle tenotomy for treatment of common extensor tendinosis in the elbow. J Ultrasound Med.

[17] Middleton K.K., Barro V., Muller B., Terada S., Fu F.H. (2012). Evaluation of the effects of platelet-rich plasma (PRP) therapy involved in the healing of sports-related soft tissue injuries. Iowa Orthop J.

[18] Rupe M.W., Fleury I.G., Glass N., Kruse R., Buckwalter V.J. (2023). Efficacy of ultrasonic tenotomy and debridement and platelet-rich plasma injections for lateral elbow tendinopathy. J Hand Surg Glob Online.

[19] Sanders T.L., Maradit Kremers H., Bryan A.J., Ransom J.E., Smith J., Morrey B.F. (2015). The epidemiology and health care burden of tennis elbow: a population-based study. Am J Sports Med.

[20] Seng C., Mohan P.C., Koh S.B., Howe T.S., Lim Y.G., Lee B.P. (2016). Ultrasonic percutaneous tenotomy for recalcitrant lateral elbow tendinopathy: sustainability and sonographic progression at 3 years. Am J Sports Med.

[21] Shapiro L.M., Welch J.M., Zhuang T., Fogel N., Hand Surgery Quality C., Ruch D.S. (2023). The use and downstream associations of magnetic resonance imaging for lateral epicondylitis. J Hand Surg Am.

[22] Shim J.W., Lee J.S., Park Y.B., Cho H.C., Jung H.S. (2022). The effect of leucocyte concentration of platelet-rich plasma on outcomes in patients with lateral epicondylitis: a systematic review and meta-analysis. J Shoulder Elbow Surg.

[23] Sims S.E., Miller K., Elfar J.C., Hammert W.C. (2014). Non-surgical treatment of lateral epicondylitis: a systematic review of randomized controlled trials. Hand (N Y).

[24] Steinmann S., Pfeifer C.G., Brochhausen C., Docheva D. (2020). Spectrum of tendon pathologies: triggers, trails and end-state. Int J Mol Sci.

[25] Wong A.K., Swami P.N., Reed T.F., Bitterman A., Grande D.A. (2018). Efficacy and safety of a percutaneous tenotomy system for debridement of tendinopathic tissues. J Long Term Eff Med Implants.

[26] Zhou H., Zhuang Z.X., Sun Y.Q., Chen Q., Zheng X.Y., Liang Y.T. (2019). Changes in DNA methylation during epigenetic-associated sex reversal under low temperature in Takifugu rubripes. PLoS One.

[27] Zhu J., Hu B., Xing C., Li J. (2008). Ultrasound-guided, minimally invasive, percutaneous needle puncture treatment for tennis elbow. Adv Ther.

